# Supplementary material for: The Role of Bioactive Peptides in Diabetes and Obesity
Source: Foods. 2021 Sep 18;10(9):2220. doi: 10.3390/foods10092220 (PMC8469013; doi:10.3390/foods10092220)
Supplement: Supplementary file 1 [file foods-10-02220-s001.zip › foods-1300697-SI.pdf]

Role of Bioactive Peptides on Diabetes and Obesity

Supplementary Files

Table S1. In vitro studies of egg-derived hydrolysates/peptides and their effects related to diabetes and obesity

References	Aims	Hydrolysis	Main findings	Additional assays	List of Peptide sequence derived from egg
[1]	Investigated multiple biological properties of peptides	Pepsin (120 min)	Three out of four peptides decreased ACE, α-glucosidase and DPP-IV activities. The peptides presented antioxidant and ion chelating activity.	DPPH radical scavenging: all peptides tested presented radical scavenging properties in the range of from 1.5 to 2.3 μM Trolox eq/mg	YINQMPQKSRE YINQMPQKSREA VTGRFAGHPAAQ YIEAVNKVSPRAG QPF
[2]	Investigated the inhibitory activity of hydrolysates against α-glucosidase and α-amylase and identifies peptides	Alcalase (180 min)	Peptides from EW decreased the levels of α-glucosidase but not α-amylase.	N/A	Ovotransferrin RVPSLM TPSPR DLQGK AGLAPY Ovalbumin RVPSL DHPFLF HAGN WIGLF
[3]	Investigated multiple biological properties related to metabolic syndrome in RAW 264.7 cells are monocyte/macrophage-like cells, originating from Abelson leukemia virus transformed cell line derived from BALB/c mice. These cells are being described as an appropriate model of macrophages. They are capable of performing pinocytosis and	Alcalase Flavourzyme Neutrase Trypsin Pepsin Pancreatin Peptidase Promod 144P (0, 2, 4, 8, 12, 24, 36 and 48 h)	Pepsin hydrolysate decreased ACE, and peptidase hydrolysate decreased the levels of ROS, C.HOL and IL-6.	Peptidase hydrolysate (24 h) exhibited hypocholesterolaemic activity with a value of 0.259 ± 0.01 mmol bound/mg protein.<br><br>The ORAC test result was equal to 1099.9 ± 0.6 μmol Trolox/g protein.	Peptidase hydrolysate (24 h) LPDEVSG DDNKVED GVDTKSD IESGSVEQA GGLVVT<br><br>Pepsin hydrolysate (8 h) FRADHPPL FSL SALAM YQIGL RADHPFL IVF YAEERYPIL YRGGLEPINF RDILNQ ESIINF

	phagocytosis				
[4]	Investigated the effect of hydrolysate on the differentiation, insulin signalling and inflammation markers of preadipocytes in 3T3 F442A are clonal sublines isolated from 3T3 mouse embryonic fibroblasts, and can be differentiated to adipocytes.	Thermoase (90 min) + pepsin (180 min)	Increased intracellular lipid accumulation and the levels of adiponectin, PPAR-γ, C/EBPα, ↑ p-ERK 1/2, p-IRβ and p-IRS-1, decreased COX-2- and TNF-α-mediated C-Jun phosphorylation and increased p-AKT after insulin treatment	Pepsin hydrolysate (8 h) exhibited hypocholesterolaemic activity equal to 0.154 ± 0.011 mmol bound/mg protein and an ORAC test result of 574.2 ± 4.0 μmol Trolox/g protein.<br><br>E.W.H. at 2.5, 5 and 10 mg/mL increased PPAR-γ expression in a dose-dependent manner.	ERYPIL VFKGL WEKAFKDED QAMPFRVTEQE
[5]	Studied the effect of specific ACE inhibitory peptides on insulin resistance induced by Ang-II and their mechanisms of action in muscular cells (The L6 myogenic line was isolated originally by Yaffe from primary cultures of rat)	N/A	IRW prevented the decrease in glucose uptake induced by Ang-II, normalized the serine phosphorylation of IRS and GLUT4 expression and increased the p-AKT level. IRW decreased AT1R, had no effect on AT2R and decreased ROS and NADPH activity. IQW and LPK peptides only exhibited antioxidant activity.	N/A	Ovotransferrin<br>IRW<br>IQW<br>LPK

Abbreviations: ACE, angiotensin-converting enzyme; Ang-II, angiotensin II; DPP IV, dipep-tidyl peptidase IV; EW, egg white; IRS-1, insulin receptor substrate 1; IRS, insulin receptor; IRβ, insulin receptor β; COX-2, cyclooxygenase 2; PPARγ, peroxisome prolifera-tor-associated receptor gamma;

C/EBP-α, CAAT/enhancer binding protein alpha; AKT, protein kinase B; ERK1/2, extracellular signal regulated kinase 1/2; TNF-α, tumour necrosis factor alpha; DPPH, 1,1-diphenyl-2-picrylhydrazyl; ROS, reactive oxygen species; CHOL, cholesterol; IL-6, interleukin 6; GL9UT4, glucose transporter 4; AT1R, angiotensin II type 1 receptor; AT2R, angiotensin II type 2 receptor; ↑ enhanced/stimulated; ↓ reduced/inhibited.

Table S2. In vivo (rodent) studies of egg-derived hydrolysates/peptides and their effects related to diabetes and obesity

References	Aims	Hydrolysis	Treatment details	Food intake and body weight (BW)	Blood/faeces/urine analysis	Tissue analysis	Main findings
[6]<br><br>Zucker obese rats	Assessed the effect of the hydrolysate NWT-03 on renovascular damage	Alcalase (6 h)	Aqueous NWT-03 (1 g/kg/day) 15 weeks	Food intake—not given B.W.—no effect	No effect on blood glucose, insulin, HBA1C, cholesterol and F.F.A. levels. Increased the level of GLP-1 only by VIL. URINE: reduced the MDA levels and decreased albuminuria.	KIDNEY: decreased inflammatory interleukins (IL-1β, IL-13) and TNF-α, improved F.G.S., decreased expression of α-SMA and increased TXA2R expression.	NWT-03 treatment induced no changes in the diabetic profile of the rats and decreased renovascular damage.
[7]<br><br>Wistar rats	Investigated the effect of EW and EWH on fat metabolism and TG content in non-adipose tissues	Protease (duration not specified)	Casein (297 g/kg), EWH (394 g/kg), EW (286 g/kg), 8 weeks	Food intake was decreased by EWH and even more greatly reduced by E.W.<br>BW was decreased by EW	EWHvs casein: EWH decreased the TG levels, ALP activity and FFA<br>EW vs E.WH: EWH decreased HDL-CHOL and FFA levels and increased total-CHOL levels.<br>FECES, EWH vs. casein: E.W.H. increased CHOL excretion.<br>EW vs EWH: EW increased TG, TBA and CHOL excretion.	EWH vs. casein: Similar results were found for all parameters, with the exception of a decrease in fat mass. MUSCLE: increase in mass, and decreases in the SCD index, TG content and G6PDH activity. LIVER: Decreases in CHOL., TG and SCD index. EW vs. EWH: Similar results were found with all parameters, with the exception of an increased mass and a decreased S.C.D. MUSCLE: E.W. decreased the SCD (LIVER).	EW and protease EWH decrease the fat content in adipose and non-adipose tissues, inhibit enzymes involved in lipogenesis and increase muscular mass and lipid excretion.

[8] Goto-Kakizaki rats	Feeding trial with EWH to study the fat and glucose contents in diabetic and normal rats	Protease (duration not specified)	Casein (200 g/kg) + EWH (267 g/kg), 6 weeks	No differences in food intake. BW was decreased by EWH	Decreases in glucose, HOMA-IR, and the SCD index were observed. No difference was detected in any of the other parameters tested.	MUSCLE: Decrease in TG and no effect on SCD LPL, FAS and G6PDH. LIVER: No chance in TG and decrease in the SCD index. Liver, adipose tissue and muscle: similar weight.	The treatment improved the blood glucose levels and HOMA-IR but not insulin secretion and also decreased the TG content in muscle and lipid accumulation in tissues.
Wistar rats			Casein (200 g/kg) + EWH (267 g/kg), 6 weeks	No differences in food intake and BW	No difference in any of the parameters tested (glucose, insulin, HOMA-R, HOMA-P, TG, NEFA, TC, HDL-CHOL, non-HDL-CHOL, adiponectin and SCD index)	MUSCLE: Decrease in SCD but no effect on LPL, FAS and G6PDH. LIVER: No effect on and decrease in the SCD index. Liver, adipose tissue and muscle: similar weight	The treatment decreased the lipid content in muscle.
[9] Zucker obese rat	Demonstrate the effects of EWH related to obesity, lipid metabolism, inflammation and oxidative stress	Pepsin (8 or 14 h)	Aqueous EWH (750 mg/kg/day) 12 weeks	No difference in food intake and BW regardless of the hydrolysate	$\downarrow$TNF-α, FFA and adiponectin, MDA No changes in blood TG and CHOL	ADIPOSE TISSUE: Decrease in weight but no changes in histology. LIVER: decreased steatosis and increase in G.S.H. Similar kidney and liver weights. Longer duration of hydrolysis negated the effects.	The treatment decreased fat accumulation, improved hepatic steatosis and dyslipidaemia, and decreased inflammatory and oxidative stress markers in plasma.

[10]<br><br>Wistar rats	Studied the effect of EW and low-allergenic EWH on fat accumulation	Protease (duration not specified)	Equicaloric diets, casein (297 g/kg), EWH (394 g/kg), E.W. (286 g/kg), 8 weeks	No difference in food intake and body weight between the three groups.	EWH vs. casein: decreases in total CHOL and ALP and similar glucose, TG, NEFA, HDL-CHOL, non-HDL-CHOL, HOMA-β and insulin levels.<br>EW vs. EWH: similar results were obtained for all parameters.<br>FECES, EWH and EW vs. casein: increases in TG, CHO and TBA.	EWH vs. casein: similar results in all parameters with the exception of decreases in weight, TG, NEFA, and SCD index.<br>LIVER: decrease in TG<br>MUSCLE, EWH vs. E.W.: similar results in all parameters with the exception of increases in G6PDH activity (muscle) and S.C.D. (adipose tissue) and decrease in F.A.S. (liver) after E.W.H. treatment.	The treatment decreased fat accumulation in non-adipose tissues and the intestinal absorption of lipids by increasing lipid excretion. Similar results were obtained with EW and EWH, but E.W.H. was less allergenic.
[11]<br><br>Obese Zucker rats	Observed the effect of EWH on the gut microbiota of rats	Pepsin (8 h)	Aqueous EWH (750 mg/kg/day), 12 weeks	Food intake: N/A<br>BW: no difference	FECES: decreases in lactate and SCFA; the abundances of *Lactobacillus/Enterococcus* and *C. leptum* were similar to those in the lean control	N/A	The treatment partially reverted the dysbiosis present in obese Zucker rats.

Abbreviations: EWH, egg white hydrolysate; FFA, free fatty acid; MDA, malondialdehyde; EW, egg white; TG, triglyceride; CHO, cholesterol; ALP, alkaline phosphatase; TBA, total bile acid; SCD, stearoyl CoA desaturase; NEFA, non-esterified fatty acid; FGS., focal glomerulosclerosis; AST, aspartate aminotransferase; ALT, alanine aminotransferase; G6PDH, glucose 6-phosphate dehydrogenase; LPL, lipoprotein lipase; FAS, fatty acid synthase; TNF-α, tumour necrosis factor alpha; α-SMA, smooth muscle alpha-actin; VIL, vildagliptin; HOMA-R, homeostasis model assessment of insulin resistance; HOMA-β, homeostasis model assessment of insulin secretion; GSH, reduced glutathione; HBA1C, glycated haemoglobin A1C; GLP-1, glucagon-like peptide-1; TXA2R, thromboxane A2 receptor; SCFA, short-chain fatty acid; WK, week; ↑ enhanced/stimulated; ↓ reduced/inhibited.

Table S3. In vitro studies of soy-derived hydrolysates (S.H.)/peptides and their effects related to diabetes and obesity

**References**	**Aims**	**Hydrolysis**	**Outcomes**	**Main findings**	**Peptide sequence**
Enzymatic activity of soy-specific peptides					
[12]	Verified that soy peptide inhibits DPP-IV in vitro and identified the regions of interactions	Pepsin- and/or pancreatin-synthetized peptides	Only IAVPTGVA decreased DPP-IV activity. The regions of interaction were in the N-termini of Glu205 and Glu206 and the C-terminus of Arg358; the peptide has a proline flanked by valine in the fourth N-terminal residue, which predicts the interaction with DPP-IV.	The soy peptide IAVPTGVA decreased DPP-IV activity in vitro. YVVNPDNDEN and YVVNPDNNEN did not show activity against DPP-IV.	IAVPTGVA<br>YVVNPDNDEN<br>YVVNPDNNEN
[13]<br><br>3T3-L1 adipocytes	Isolated and identified peptides from soy hydrolysate with lipolytic activity	Flavourzyme 1% (125 min)	Three peptides increased glycerol release. After in vitro simulated GI digestion, the V.H.V.V. capacity was not affected, and ILL and L.L.L. exhibited attenuated lipolytic activity.	Soy peptides increased lipolysis in 3T3-L1 adipocytes and were not or only slightly affected by GI enzymes.	ILL<br>LLL<br>VHVV
[14]<br><br>Human HepG2 cells	Verified that soy peptides modulate glucose metabolism	Trypsin- or pepsin-synthetized peptides	All three peptides increase the p-AKT level, GLUT 4 and GLUT 1 mRNA expression and glucose uptake and decrease GSK3 activation. (IAVPTVGVA > IAVPGEVA > LPYP). IAVPGEVA and IAVPTVGVA induced greater increases in GLUT1 , LPYP and GLUT4 mRNA	Soy peptides modulate glucose metabolism and increase glucose uptake in liver cells through activation of the AKT and AMPK pathways.	IAVPGEVA IAVPTGVA<br>LPYP

			expression.		
[15]<br><br>RAW 264.7 macrophages and 3T3-L1 adipocytes	Demonstrated the mechanism of transport of soy peptides into adipocytes and evaluated the TNF-α induced inflammation and insulin response	Synthetized peptide	FLV peptide decreased the TNF-α, MCP-1 and IL-6 levels in the cocultured cell lines (macrophages + adipocytes). FLV decreased the TNF-α-induced phosphorylation of JNK and the phosphorylation of IKK, decreased the degradation of IκBα and ameliorated TNF-α-induced insulin resistance in adipocytes (↑ p-IRS-1, p-AKT) PepT2 > PepT1 expression in adipocytes is increased by L.P.S. and TNF-α.	FLV is transported into adipocytes mainly through PepT2 action, and FLV can decrease the inflammatory and insulin resistance states linked to obesity mainly by decreasing TNF-α-induced inflammatory pathways	N/A
[15]<br><br>3T3-L1 adipocytes and RAW 264.7 macrophages	Studied the effect of S.H. on lipid accumulation and inflammation	Alcalase (3 h) or<br>pepsin + pancreatin (3 h each)	In 3T3-L1 cells, alcalase SH decreases lipid accumulation and LPL and FAS mRNA expression. Simulated GI digestion did not reduce the bioavailability of alcalase SH Compared with pepsin + pancreatin SH, alcalase SH decreased LPL and FAS mRNA expression to a higher extent before and after GI digestion. In RAW cells, alcalase SH decreased LPL-induced nitrite formation, iNOS and COX-2 protein	SH decreased lipid accumulation and inflammatory marker expression, even after simulated GI digestion. The downregulation of LPL and FAS partially explains the mechanism of action. Higher concentration of β-conglycin in the hydrolysate are related to higher activity in vitro.	N/A

			expression, and PGE2 production. In 3T3-L1 cells, pepsin + pancreatin S.H. decreased lipid accumulation and L.P.L. but not FAS mRNA expression.		
[16]<br><br>3T3-L1 adipocytes	Investigated the effect of germinated vs. ungerminated S.H. on fat metabolism in adipocytes and assessed the interaction with soy phytochemicals	Pepsin + pancreatin (duration not specified)	At a concentration > 1 mg/mL, the treatment decreased cell viability during the differentiation process (10 days incubation), but not during 24 h of exposure. S.H. with and without phytochemicals reduced lipogenesis, and a higher germination time was correlated to a greater reduction in lipogenesis. Lipolysis was reduced in a dose-dependent manner only by the treatment with S.H. without phytochemicals.	Germination changed the amino acid composition in the S.H. and interfered with the responses. Overall, S.H. reduced the number of adipocytes during the differentiation process and increased lipolysis in mature adipocytes.	N/A
[17]	Observed the effects	Peptic hydrolysate	During adipocyte	SH increased adipocyte	N/A

3T3-L1 pre-adipocytes	of soybean peptic hydrolysate on adipocyte differentiation	(duration and enzymes not specified)	differentiation, SH dose-dependently increased lipid accumulation, aP2 mRNA expression, adiponectin mRNA expression and secretion, PPAR-γ mRNA and protein expression, glucose uptake, and GLUT4 mRNA expression.	differentiation via the PPAR-γ pathway and increased glucose uptake during the differentiation process.	
[18]<br><br>L6 skeletal muscle cells	Verified the potential of EDUF to concentrate soy peptides and identified the mechanism of action of those peptides	Pepsin (45 min) + pancreatin (120 min)	In the initial hydrolysate, anionic and cationic peptides increased glucose uptake. Only the peptides increased the p-AMPK level.	Anionic and cationic soy increases glucose uptake and AMPK phosphorylation in L6 skeletal muscle cells in vitro.	N/A

Abbreviations: SH, soy hydrolysate; Ap2, adipocyte fatty acid-binding protein; IRS-1, insulin receptor substrate 1; COX-2, cyclooxygenase 2; PPARγ, peroxisome proliferator-associated receptor gamma; AKT, protein kinase B; TNF-α, tumour necrosis factor alpha; LPL, lipoprotein lipase; FAS, fatty acid synthase; GLUT4, glucose transporter 4; GLUT1, glucose transporter 1; GI, Gastrointestinal; iNOS, inducible nitric oxide synthase; PGE2, prostaglandin E2; AMPK, activated protein kinase; JNK, c-Jun N-terminal kinase; IKK, IκB kinase; PepT2, peptide transporter 2; PepT1, peptide transporter 1; IL-6, interleukin 6; DPP-IV, dipeptidyl peptidase IV; MCP-1, monocyte chemoattractant protein-1; L.P.S., lipopolysaccharide; GSK3, glycogen synthase kinase 3; ↑ enhanced/stimulated; ↓ reduced/inhibited.

References

1. Pan, D.; Luo, Y.; Tanokura, M.J.F.C. Antihypertensive peptides from skimmed milk hydrolysate digested by cell-free extract of Lactobacillus helveticus JCM1004. *Food Chem.* **2005**, *91*, 123–129.
2. Nasri, M. Protein hydrolysates and biopeptides: Production, biological activities, and applications in foods and health benefits. A review. *Adv. Food Nutr. Res.* **2017**, *81*, 109–159.
3. Eckert, E.; Lu, L.; Unsworth, L.D.; Chen, L.; Xie, J.; Xu, R. Biophysical and in vitro absorption studies of iron chelating peptide from barley proteins. *J. Funct. Foods* **2016**, *25*, 291–301.
4. Zambrowicz, A.; Pokora, M.; Setner, B.; Dąbrowska, A.; Szołtysik, M.; Babij, K.; Szewczuk, Z.; Trziszka, T.; Lubec, G.; Chrzanowska, J. Multifunctional peptides derived from an egg yolk protein hydrolysate: Isolation and characterization. *Amino Acids* **2015**, *47*, 369–380.
5. Yu, Z.; Yin, Y.; Zhao, W.; Yu, Y.; Liu, B.; Liu, J.; Chen, F. Novel peptides derived from egg white protein inhibiting alpha-glucosidase. *Food Chem.* **2011**, *129*, 1376–1382.
6. Jahandideh, F.; Chakrabarti, S.; Davidge, S.T.; Wu, J. Egg white hydrolysate shows insulin mimetic and sensitizing effects in 3T3-F442A pre-adipocytes. *PLoS ONE* **2017**, *12*, e0185653.

7. Son, M.; Chan, C.B.; Wu, J. Egg white ovotransferrin-derived ACE inhibitory peptide ameliorates angiotensin II-stimulated insulin resistance in skeletal muscle cells. *Mol. Nutr. Food Res.* **2018**, *62*, 1700602.
8. Claessens, M.; Saris, W.H.; van Baak, M.A. Glucagon and insulin responses after ingestion of different amounts of intact and hydrolysed proteins. *Br. J. Nutr.* **2008**, *100*, 61–69.
9. Wang, Y.; Landheer, S.; van Gilst, W.H.; van Amerongen, A.; Hammes, H.-P.; Henning, R.H.; Deelman, L.E.; Buikema, H. Attenuation of renovascular damage in Zucker diabetic fatty rat by NWT-03, an egg protein hydrolysate with ACE-and DPP4-inhibitory Activity. *PLoS ONE* **2012**, *7*, e46781.
10. Ochiai, M.; Matsuo, T. Effect of egg white and its hydrolysate on stearoyl-CoA desaturase index and fat accumulation in rat tissues. *Int. J. Food Sci. Nutr.* **2014**, *65*, 948–952.
11. Ochiai, M.; Kuroda, T.; Matsuo, T. Increased muscular triglyceride content and hyperglycemia in Goto-Kakizaki rat are decreased by egg white hydrolysate. Int. J. Food Sci. Nutr. 2014, 65, 495–501.
12. Lammi, C.; Zanoni, C.; Arnoldi, A.; Vistoli, G. Peptides derived from soy and lupin protein as dipeptidyl-peptidase IV inhibitors: In vitro biochemical screening and in silico molecular modeling study. *J. Agric. Food Chem.* **2016**, *64*, 9601–9606.
13. Tsou, M.-J.; Kao, F.-J.; Lu, H.-C.; Kao, H.-C.; Chiang, W.-D. Purification and identification of lipolysis-stimulating peptides derived from enzymatic hydrolysis of soy protein. *Food Chem.* **2013**, *138*, 1454–1460.
14. Kwak, S.-J.; Kim, C.-S.; Choi, M.-S.; Park, T.; Sung, M.-K.; Yun, J.W.; Yoo, H.; Mine, Y.; Yu, R. The soy peptide Phe–Leu–Val reduces TNFα-induced inflammatory response and insulin resistance in adipocytes. *J. Med. Food* **2016**, *19*, 678–685.
15. Martinez-Villaluenga, C.; Dia, V.P.; Berhow, M.; Bringe, N.A.; Gonzalez de Mejia, E. Protein hydrolysates from β-conglycinin enriched soybean genotypes inhibit lipid accumulation and inflammation in vitro. *Mol. Nutr. Food Res.* **2009**, *53*, 1007–1018.
16. de los Monteros, L.G.-E.; Ramon-Gallegos, E.; Torres-Torres, N.; Mora-Escobedo, R. Effect of germinated soybean protein hydrolysates on adipogenesis and adipolysis in 3T3-L1 cells. *Plant. Foods Hum. Nutr.* **2011**, *66*, 355–362.
17. Goto, T.; Mori, A.; Nagaoka, S. Soluble soy protein peptic hydrolysate stimulates adipocyte differentiation in 3T3-L1 cells. *Mol. Nutr. Food Res.* **2013**, *57*, 1435–1445.
18. Roblet, C.; Doyen, A.; Amiot, J.; Pilon, G.; Marette, A.; Bazinet, L. Enhancement of glucose uptake in muscular cell by soybean charged peptides isolated by electrodialysis with ultrafiltration membranes (E.D.U.F.): Activation of the A.M.P.K. pathway. *Food Chem.* **2014**, *147*, 124–130.
